# A Review of Intelligent Driving Style Analysis Systems and Related Artificial Intelligence Algorithms

**DOI:** 10.3390/s151229822

**Published:** 2015-12-04

**Authors:** Gys Albertus Marthinus Meiring, Hermanus Carel Myburgh

**Affiliations:** Department of Electrical, Electronic and Computer Engineering, University of Pretoria, Private Bag X20, Hatfield, Pretoria 0028, South Africa; gam.meiring@gmail.com

**Keywords:** driving style, driver behaviour, artificial intelligence, machine learning, driver safety, road accident, driver identification

## Abstract

In this paper the various driving style analysis solutions are investigated. An in-depth investigation is performed to identify the relevant machine learning and artificial intelligence algorithms utilised in current driver behaviour and driving style analysis systems. This review therefore serves as a trove of information, and will inform the specialist and the student regarding the current state of the art in driver style analysis systems, the application of these systems and the underlying artificial intelligence algorithms applied to these applications. The aim of the investigation is to evaluate the possibilities for unique driver identification utilizing the approaches identified in other driver behaviour studies. It was found that Fuzzy Logic inference systems, Hidden Markov Models and Support Vector Machines consist of promising capabilities to address unique driver identification algorithms if model complexity can be reduced.

## 1. Introduction

Driving- and road safety are current and growing problems with global dimensions. According to the global status report on road safety conducted by the World Health Organisation (WHO) in 2013, 1.24 million traffic-related fatalities occur annually worldwide, currently the leading cause of death for people aged 15–29 years [1]. As a result of the increased need for mobility in developing countries the continuous expansion of vehicle manufacturing is evident. The evolution of the global vehicle fleet causes an infrastructure backlog [2], which in turn is responsible for increased traffic safety risks and accident prevalence. Driver assistance and safety awareness programmes have been an area of focus to minimise road safety incidents, and since the WHO launched their “Decade of Action in Road Safety (2011–2020)” programme, a remarkable improvement in road safety has been noticeable [3]. Despite the growth of 15% in the annual number of registered vehicles from 2007 to 2013, the annual fatalities remained stable in the region of 1.2 million over the same period [4]. However, a saturation in the number of fatalities is not good enough and a reduction should be observed instead. High priority is given to traffic safety improvements by government agencies and major automobile manufacturers across the globe to address this problem, and innovation in driver assistance is currently in demand.

The contribution of human behaviour towards traffic accidents is an important area of interest in the remedial attempts to address the global road safety problem. A clear distinction needs to be made between errors and violations to ameliorate traffic accidents, due to the different psychological origins of these components of human behaviour, as well as the dissimilar forms of remediation [5]. The Driver Behaviour Questionnaire (DBQ) is a metric widely used in driver behaviour studies over the past few decades, and focus falls on the correlation between the sub-components of both violations and errors, and the degree to which these components are related to crash involvement [6]. Around 12,000 novice drivers were tested repeatedly with the DBQ in the first six months after they passed their driver’s licence tests and analysis of the data accumulated from this experiment proved the trustworthiness of the DBQ as a driver behaviour measure in traffic accident prediction [6]. The integrity and validity of the DBQ has been confirmed [7]. The statistical correlation between driver behaviour and crash involvement is particularly related to individual variability associated with numerous parameters such as age, gender and geographic locations [8]. Interestingly, DBQ results shown that violations declined with age as opposed to errors and the prevalence of violation is higher in men than in woman [5,7]. Results obtained using the DBQ are used as predictor of self-reported traffic accidents, but the question regarding the trustworthiness of the metric is raised due to the reporting bias and the non-real-time recording of the measurement, which serves a remedial instead of a reactive purpose, and therefore real-time evaluation of driver behaviour information and patterns are investigated as a solution to mitigate risk through driver assistance and early warning of predicted dangerous situations.

One of the primary focusses of this investigation is to analyse research in the area of driver behaviour solutions in order to address road safety problems. This is achieved through the identification and understanding of the relationship between driver behaviour and road safety, and the classification of drivers using their propensity to risk [9]. The relationship between the driver and the vehicle control, and the sensitivity of the driver to complex driving environments or situations (e.g., weather, traffic) are major contributing factors towards traffic accidents [10]. According to [11], traffic accident involvement is more closely related to human judgement and decision-making than the mere inability to control the vehicle, and therefore, the focus of driver behaviour and decision-making patterns became a popular research area in road-safety applications. The authors in [11] approached the study of decision-making in two different ways: To analyse the beliefs and values that constitute the decision process, and to evaluate the style of decision-making based on individual habits and personality traits. The second approach proved to be representative and measurable in applications through the classification of drivers into predefined driving styles.

The aim of the study is to investigate the possibilities of utilizing the aforementioned driving style information or intelligence, in order to construct driver profiles that might be able to uniquely represent and identify one driver from another in the same vehicle based upon their driver behaviour differences in similar situations. Therefore, four main driving styles were distinguished from the various interpretations of driving styles found in the literature based on the measurable differences between them. This is done in an attempt to move beyond the mere classification of a driver in a certain “risk profile” or “behavioural group” and to combine driving style information in different situations and phases of driving to construct a unique “driver behaviour fingerprint”. The unique identification of a driver can be useful in many commercial applications, especially in the insurance sector where a driver’s risk profile based upon his driving behaviour can be extrapolated to different environments that account for prospective personal risk, such as insurance and loan qualification, as well as insurance premium calculation and adjustment over time. Vehicle theft and dishonesty during insurance claims can also be addressed with the introduction of unique driver profile construction and accurate driver distinguishing applications. The four types of driving styles investigated in this study namely, aggressive driving style, inattentive driving style, drunk driving style and “normal” or safe driving style, were particularly identified due to the differences between the classification processes implemented, the input data analysed and the algorithms used to predict and label driver behaviour into specific driving styles. These driving styles are adopted from well-researched driver behaviour types but specific focus fell on the interpretations of [12,13,14].

Research in the areas of driver behaviour monitoring, navigation task detection, driving style recognition and prediction are constantly growing as the need for innovative, immediate and accurate solutions develop with the excel in vehicle fleet and road infrastructure industries. The body of knowledge around driver behaviour research is subdivided in this study according to differences in application and sensor platforms over a range of fields and industries. This review further categorises the driver behaviour application areas into principal groups of which methods and results are compared in order to enhance the understanding of the implementation and application of driver behaviour analysis. The artificial intelligence (AI) and machine learning (ML) algorithms applied in advanced and recent driver behaviour applications are defined properly and categorized according to their intended application in order to identify correlations between the type of algorithms applied and the applications they serve. This was performed in order to compile a scope of possible algorithms to investigate in the proposed problem of unique driver identification.

The difficulties experienced in conducting this study included the scope of the research area and the depth of investigation that were necessary, as well as the categorization of algorithms according to the respective applications they serve. The reason therefore, was due to the use of similar solutions, concepts and design architectures to a range of different industries and applications. The techniques followed and algorithmic uses differ depending on the type of input data the authors had access to and the accuracy of the data that was required in different approaches, and therefore, identifying the studies from which techniques could be adopted for further research and experimental purposes, posed further challenges. This study was further complicated due to many applications only utilizing the state of the art technologies equipped in the latest vehicles, and neglects to propose similar solution for existing vehicles with outdated technologies which might be the origin of the road safety problems. Smartphone applications or after-market products might be possible equipment to investigate further to address this complication. Research solutions and experiments are very limited to the specific problem they address and due to the great variability of driver behaviour, assumptions and results need to be substantially motivated and proven. Driver behaviour is a well-researched area with some unexplored possibilities but complications such as complexity of models and real-time data acquisition and processing need to be taken into account. Fuzzy logic systems seem to be a valuable area for initial investigation as scoring techniques can easily be extended to evaluate multiple areas of driver behaviour to infer conclusions (in the form of average scores) in a certain driving situation.

## 2. Driver and Road Safety Concerns in Developing Countries

Driver and road safety improvement programmes, for instance remedial driver education programmes, are more actively pursued in first-world countries with specific reference to available driver behaviour and driver style information. Although results from the UK Department of Transport’s report for road casualties in Great Britain for 2011 indicates that the decrease of 5% in road accident injuries and fatalities from the previous year is attributed to driver awareness campaigns [14], strong evidence opposing this claim is given in [15] in which trial reviews indicated that no impact exists of driver education on the reduction in traffic crashes or injuries. Other possibilities should therefore be investigated.

**Figure 1:**
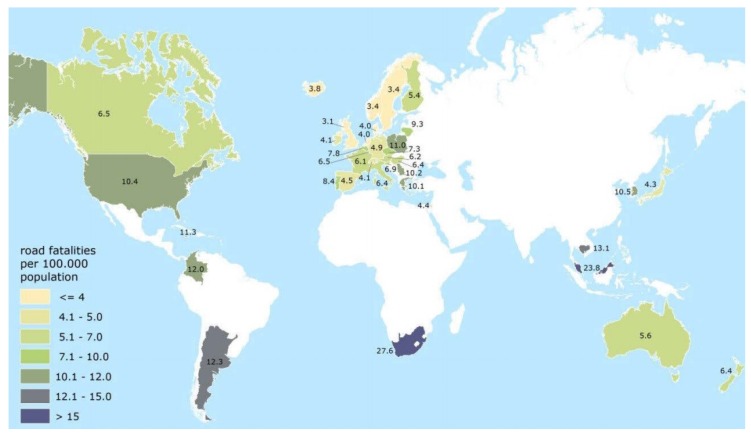
Road fatalities per 100,000 population in 2011 (International Road Traffic and Accident Database (IRTAD) annual road safety report 2013) [18].

Third-world countries especially struggle to address road and vehicle safety concerns due to infrastructural and economic challenges. These challenges are introduced by the rapid growth of vehicle fleets, increased driver carelessness and neglected law enforcement in these countries. The Ministry of Works of Malaysia provides evidence of this statement in research findings, attributing 41% of vehicle collisions as the direct result of driver carelessness [16]. More evidence is described in [17], that although vehicles cater for human convenience and mobility, bad driving practices, traffic congestion and vehicle maintenance negligence threaten peoples’ lives and property. The International Transport Forum’s (ITF) annual road safety report for 2013 ranked South Africa as the country with the highest number of road fatalities, from the 37 countries evaluated, with a road fatality ratio of 27.6 deaths per 100,000 inhabitants in 2011, as opposed to developed countries such as North America with 10.4, Australia with 5.6, and the UK with 4.1 [18]. The results for this sample is shown in Figure 1. It is apparent that an immense void exists in the market in South Africa and other developing countries, with ample opportunity to introduce and implement driver and road safety enhancement solutions through driver behaviour applications.

Vehicle accidents and road damages greatly affect economic growth. The International Road Traffic and Accident Database (IRTAD) also mentioned in the road safety report that the estimated economic cost of South Africa’s road accidents amounts to approximately 24.38 billion US dollar annually [18]. This economic burden is not limited to a single country but has become a general problem worldwide, with accident-related costs in 2011 in the US estimated to be 299.5 billion US dollar [9]. Occupational fatalities is another area that greatly influences economic growth, and according to [19] work-related accidents are the topmost individual cause of occupational fatalities in Australia. It is evident that road safety improvement is an essential responsibility of developing countries world-wide.

## 3. Driving Styles

Driver behaviour is a comprehensive term used to represent different concepts related to a driver’s driving actions and driving mannerisms which introduce endless variables. It is therefore hypothesized that by defining and extending driver behaviour into driving styles, multiple actions of a driver in a specific category of driving can be represented and similarities in measurements and behaviour trends can be identified more accurately due to the definitive and measurable nature of driving styles. If high accuracy of driver behaviour prediction can be obtained through the application of driving styles, it will greatly contribute to the purpose of this study. The driving styles identified to investigate further are “normal” or safe driving style, aggressive driving style, inattentive driving style and drunk driving style and the next section will elaborate on the current applications of these driving styles.

### 3.1. Normal / Safe Driving Style

In driver style applications the main goal is to detect behaviour out of the ordinary [16], and for learning and classification techniques, a reference driving style should be defined from which deviation can be identified. In this study online driver behaviour classification into pre-set driving styles is investigated. Aggressive, inattentive and drunk driving styles are styles of driving that deviate from the norm and expected behaviour of a driver. It is therefore necessary to define a driving style that can represent a driver’s normal behaviour in order to identify behaviour that diverges from the norm. For instance, aggressive driving is classified when the driver’s behaviour proves to be more aggressive than the normal state of driving recorded for the specific driver based on his behaviour during the training of the classifier.

This approach is not a new initiative and are followed in many driving style analysis studies and referred to as either “normal driving”, “safe driving”, “typical driving” or “non-aggressive” driving. An euclidean norm was evaluated on accelerometer data in [13] in order to use the deviations from the average of this norm to classify the driving behaviour in different classes, in this case Below Normal (BN), Normal (N), Aggressive (A), Very Aggressive (VA). In [16], speed and the change in lateral position were used as parameters in the driving style detection algorithm and deviations from normal driving were used to identify abnormal driving events. In order to enable for differentiation with high selectivity, a discrete scale with different levels was used to distinguish different driving styles using the deviations from the norm as classifier parameter in [20].

The normal driving style may vary greatly between drivers depending on various variables, such as the AI algorithms implemented, the vehicle used and the behaviour of the driver during the training period of the classifier. The study is not focussed on identifying a style of driving which should correlate between drivers, but rather the deviation of driver behaviour based on the recorded driving history for an individual. A “safer driver” can easily be confirmed with accrued driving experience and increased recorded driver history as sufficient driving behaviour history data will improve machine learning classification.

For road safety enhancement purposes it can be advantageous to determine a safe or ideal driving style to which a driver can adapt his behaviour, once characteristics of other risky driving styles such as aggressive driving, inattentive driving or drunk driving have been identified.

### 3.2. Aggressive Driving Style

Risky driving and to a large extent risk induced through aggressive driving, has been identified as a major contributing behavioural characteristic that influences road safety for the driver personally, as well as for other drivers travelling in close proximity to the aggressive driver. A study conducted by the American Automobile Association Foundation for Traffic Safety in 2009 found that “as many as 56% of deadly crashes between 2003 and 2007 involve one or more unsafe driving behaviors typically associated with aggressive driving” [21,22].

An aggressive driving style is a behavioural pattern or classification of a driver which is associated with risky speeding profiles (irregular, instantaneous and abrupt changes in vehicle speed), improper vehicle position maintenance (quick changes in lateral vehicle position) and inconsistent or excessive acceleration and deceleration (harsh take-off and braking) [13]. Another definition of an aggressive style of driving is driver behaviour that intentionally increases the risk of collision and can be due to driver impatience, annoyance, hostility or an attempt to minimise travelling time [9]. The characteristics included in the classification of an aggressive driver depend on the application, and the learning or classification algorithm used to place a driver in a certain driving style, but more examples of aggressive behaviour may include tailgating and improper lane changes.

The relationship between health risks and aggressive driving also resulted in meticulous research in the field of psychology, as it is believed that aggressive driving is a dysfunctional arrangement of social behaviours and a public safety threat. In [23], a questionnaire assessment instrument was designed to measure aspects of aggressive driving on a scale called the aggressive driving behavior scale (ADBS). The ADBS was recently extended [24], to a Prosocial and Aggressive Driving Inventory (PADI), which distinguishes between safe and unsafe driving behaviours. It was found that aggressive driving corresponds to a tendency of increased competitiveness, sensation or adrenalin pursuit, and hostility, and a negligence of conscientiousness, agreeableness, and tolerance. The differences in driving styles and behavioural patterns among drivers with unique personality traits and psychological frameworks are invaluable attributes to pursue in further studies related to unique driver identification.

### 3.3. Inattentive Driving Style

Driver behaviour which indicates regular inattention to driving actions and necessary observations to complete the driving task is classified as “inattentive driving style” in this study. Most probably, inattention while driving can be observed as an instantaneous deviation from normal behaviour with the immediate following of sudden driver actions to correct behaviour resulting from inattention in the form of a reaction. An inattentive driving style differs from an aggressive driving style due to its instantaneous and sporadic nature, as opposed to an aggressive driving style which is can be observed as a pattern of misbehaviour over a period of time.

Inattention is a major contributing factor to the road safety problem introduced earlier in this report. A study named the “100-Car Naturalistic Study” [25] was designed by the National Highway Safety Administration (NHTSA) in collaboration with the Virginia Tech Transportation Institute (VTTI) to provide insight on the influence and contribution of driver behaviour immediately preceding an accident. This study was performed on 100 vehicles fitted out with surveillance and other sensor devices for a duration of a year, driven collectively for nearly 2 million miles, and accumulated 42,000 h of data from the 241 drivers. It revealed that 78% of the 82 accidents recorded, and 65% of the 761 near accidents were the direct effect of driver inattention [26]. In 2010 in the USA alone, 3092 people were killed and 416,000 injured during accidents directly attributed to driver inattention [27]. It is estimated that 13.3% of accidents can be attributed to the involvement of driver distraction and 9.7% of crashes categorised as “looked but did not see” [28].

Driver inattention is an ambiguous description, as inattention is influenced by many factors and measurable events of inattention should be identified. Therefore a breakdown of “driver inattention” is performed to enable conclusions to be drawn of the state of inattention. A recent review of the current state of the art in driver inattention monitoring classified two main categories in monitoring a driving style of inattention, namely, driver fatigue and driver distraction [29]. The categorisation of inattention is made based on the difference between distraction and the phenomenon of fatigue. Fatigue exhibits physical symptoms such as reduced performance and a subjective feeling of drowsiness, whereas distraction may be due to a number of unpredicted reasons that occur at random. One possible application of the distinction between states of inattention can include the classification of a driver as a distraction prone driver or fatigue prone driver.

#### 3.3.1. Driver Fatigue

Driver fatigue is a measurable element of inattentive driving. Most drowsy drivers will try to fight against falling asleep in an attempt to prevent causing an accident, with deviations in sequenced physiological events, and durations of these events, as compared to similar non-sleepy behaviour. Symptoms observable in the event of a fatigued driver include repeated yawning, confusion, feeling depressed and irritable, delayed reactions and responses, daydreaming, lazy steering, difficulty keeping the eyes open and burning sensation in the eyes, lapses in concentration, swaying of the head, shallow breathing, racing heart beats, and the vehicle wandering between lanes or from the road [29].

A report on the influence of driver fatigue on road accidents conducted by the European Transport Safety Council (ETSC) mentioned that fatigue “concerns the inability or disinclination to continue an activity, generally because the activity has been going on for too long” [30]. It was concluded that central nervous fatigue (sleepiness) and mental fatigue (lack of energy to do anything) of the driver, were more dangerous towards driving than physical fatigue. There is a clear indication that 15%–20% of all vehicle accidents are estimated to be related to sleepiness and that fatigue driving increases the risk of an accident occurring by four to six times [31]. The prevalence of accidents are higher at night than during the day [32], which also confirms the consequences of fatigue driving on accident occurrence. Bad lighting conditions at night time naturally also contributes to this phenomenon.

#### 3.3.2. Driver Distraction

Driver distraction is defined in [29] as “a diversion of attention away from activities critical for safe driving toward a competing activity”. Potential distracting activities performed by drivers while driving may include eating or drinking, attention to a person, object or event outside the car, talking, texting or listening on a cellular phone, using in-vehicle-technologies, and distracting weather conditions (rain or mist for instance). The NHTSA classifies distractions from the viewpoint of the driver’s functionality into four categories, namely visual distraction, cognitive distraction, auditory distraction and biomechanical distraction (e.g., manually adjusting radio volume). A distracting activity can include a combination of the above categories.

Driver distraction has been playing a role in road safety since the introduction of vehicles. The problem of driver distraction is possibly increasing as technology evolves and more functionalities are introduced to modern vehicles, such as in-vehicle information systems (IVIS), for instance navigation systems, and entertainment systems [28]. Mobile technology also greatly increases the probability of causing distraction while driving. This can be noticed through laws introduced by numerous countries’ governments, prohibiting the use of mobile hand-held phones while driving.

### 3.4. Drunk Driving Style

Another measurable area of driver behaviour is the influence of intoxication on an individual’s normal driving behaviour. A clear deterioration to a whole range of tasks and behaviour is evident upon intoxication, due to intoxication having a degenerative influence on personal traits such as competence to perform a task, and reduction in self-discipline and concentration (ultimately leading to risky and out of the ordinary behaviour) [33]. A drunk driving style may include aspects of the aggressive driving style but due to the degenerative influence of alcohol the degradation of a driver’s performance from his normal driving behaviour can be identified and this information can indicate that a driver might be driving under the influence of alcohol. The inattention introduced by intoxication will be best measured as part of the inattentive driving style.

Road safety is greatly influenced by drivers that drive under the influence of alcohol. The US NHTSA have estimated the death toll caused by traffic accidents in the United States since 1966 to be more than a million people, and in 2007 and 2008 alone, 24,814 alcohol-impaired driving fatalities have been recorded and was responsible for approximately 32% of all driving fatalities for those years [34]. The presence of alcohol in a driver’s bloodstream greatly reduces the ability of the driver to operate a vehicle safely [33]. Vehicle accident occurrence proliferation is not the only result of driving under the influence (DUI), but accident severity is greatly enhanced due to the increased likelihood of injury or death from the same impact occurring with increased blood alcohol concentration (BAC) in a person’s bloodstream [35]. DUI of alcohol, is one of the prime causes of traffic accidents [36], and due to the characteristic behaviour, observable from a driver who is driving drunk, the use of alcohol intoxication detection applications are actively being investigated through various research efforts. Drunk driving has formed a part of many context-aware driver behaviour systems [14,37] and plays an important role in driver-feedback and driver assistance applications aiming to prevent accidents or to notify authorities of possible intoxicated drivers [34].

## 4. Applications Assessing Driver Behaviour

Applications using driver behaviour and driving style analysis are investigated by categorizing application that serve the same purpose together. This categorization serves invaluable advantages as it enables the identification of innovation in an area that is not saturated and extensively researched to date. It is important to note that most of the applications assessing driving style comprise of techniques that are not limited to a single driving style, and different driving styles are used interchangeably between studies, depending on the requirements of the proposed solution.

### 4.1. Driver Assistance

Driver Assistance Systems (DAS) have become increasingly popular with the advances in vehicle technology [20]. There are many reasons for this increase in popularity, most importantly road safety, as drivers are normally unaware of committing potentially dangerous actions daily [21]. Real-time analysis and auditory alerts of risk will increase a driver’s overall attentiveness and maximise safety [38]. DAS can mitigate or prevent road accidents by providing supportive information on approaching traffic in various circumstances [39]. Therefore, these DAS are specifically developed to enhance, automate and adapt vehicle systems for improved safety, enriched driving experience and improved travel comfort.

Advanced driving assistance systems (ADAS), such as lane-keeping assistance systems, forward collision warning systems and emergency braking systems, have been introduced lately by vehicle manufacturers in an attempt to address driver safety [27,38,40]. It remains true that ADAS induces another form of distraction. Driver assistance innovation is an important focus in the design, development and manufacturing of new vehicles, and includes among other functionalities automated lighting, adaptive cruise control, predictive emergency braking, navigation assistance, traffic detection and rerouting, an in-vehicle mobile device interface, driver alerts, important vehicle or weather condition notifications, and live video footage of blind spots while driving or rear view imagery during parking.

DAS and ADAS can be adapted to measure any one of the driving styles identified in this study of the driver in real-time and then provide the driver with feedback based upon driving style information. If aggressive driving style is measured for example, improved behaviour can be suggested, and when inattention and drunk driving behaviour is detected real-time feedback might prevent the driver from continuing with the current risky behaviour.

### 4.2. Drowsiness Detection

A drowsy driving style is commonly analysed using advanced solutions that make use of multiple sensor data acquisition platforms. These advanced sensors provide intelligent information on the driver’s physiological signals, which can include eye activity measures, the inclination of the driver’s face, sagging posture, heart rate monitoring, skin electric potential, and electroencephalographic (EEG) activities [37]. Many possibilities exist for monitoring drowsiness, depending on the type of sensors used, which could include steering wheel gripping force measures, lane keeping drift and instantaneous swerving patterns.

Specific research focus has fallen on sleepiness monitoring systems by dividing the techniques into three focal categories, namely driver biomedical signal based techniques, driver performance evaluation techniques based on steering behaviour, and driver visual analysis techniques using image processing and computer vision [32]. In the first category, driver biomedical evaluation can prove very effective in real-time determination of driver drowsiness, but requires for instance electrodes attached to the body of the driver. This approach is extremely invasive, and may cause irritation to the driver, regardless of the expensive installation and maintenance costs. Driver performance approaches in the second category involve variations in the lateral position of the vehicle, steering wheel angle, velocity, acceleration, as well as other controller-area-network (CAN) signals. This approach is ideal due to meaningful and easily accessible data, and has already entered commercial markets. However, numerous limitations exist for this approach which include vehicle type, driver experience and road conditions, and analysing deviations in vehicle signals might not account for a driver falling asleep on a straight road section without an explicit change in steering, heading or acceleration. This approach also finds application in monitoring and classifying other driving styles besides fatigue driving style. The third category includes techniques based on computer vision, which are natural and non-intrusive techniques that are effectively used in visual analysis systems. The reasons why these techniques are effective for drowsiness detection is due to the fact that facial appearance, head position and eye activity of a driver, are directly correlated to sleepiness and fatigue [14]. A dashboard mounted driver facing camera serves as the primary data acquisition sensor for these techniques, while the various solutions vary in the position and shot angle of these cameras as well as in the features extracted and applied in learning and classification.

### 4.3. Driver Distraction Detection

The influence of driver distraction has been investigated in driver simulation research where the driver has to perform secondary tasks while driving, such as solving trivial math problems or repeating alphanumeric strings (ALs) [41], and observations were made that indicate drastic changes in lane-changing behaviour [32], the frequency and delay of checking behaviour (check a side mirror or speedometer), and the shrinking of the visual field of the driver [19]. The classification of the driver’s state in real-time can effectively be integrated in the IVIS and ADAS technologies by identifying a distracted driver through his driving behaviour, and then communicate the information to the driver, in order to remind the driver to be more attentive to driver actions and surroundings.

#### 4.3.1. Early-Warning Applications

In the instance where probable risk while driving is determined, one way to reduce the risk is to warn the driver prior to an incident of the risk, which can either be distraction, aggression, drunk driving or external risk originating from another vehicle or object. Such solutions and applications already exist, and systems aimed at early detection and alert of dangerous vehicle manoeuvres typically related to drunk driving, proved highly efficient using a smartphone as the sensory, processing and feedback platform [17]. Reliability assessment with real-time feedback was successfully implemented in [10], which predicts future driving events by assessing the driver’s driving style pattern history.

Another approach is the grading of a driver on a “safety scale” based on driving style and driver behaviour analysis, and providing real-time information and feedback to assist in order to improve consciousness and promote safe driving [8]. The method of scoring a driver’s performance or risk is elaborated on further in other applications in particular insurance practices [27,42,43].

A computer-vision system was designed in [4] that not only observes activities and risk outside the vehicle, *i.e*., instance detecting and tracking roads, and avoid hitting obstacles or pedestrians, but simultaneously look inside the vehicle to monitor the awareness of the driver and predict the driver’s intentions (predicted driving actions to follow) and state of mind. This system considers all three components that influence driving namely the environment, vehicle and driver, and in order to promote safe driving, alerts the driver of risk originating from any one of these components. More computer-vision alert systems exist such as *DriveSafe* [27], a driver safety application for iPhones that identifies inattentive driving styles and provides relevant feedback to drivers, and *SmartV* [36], an intelligent vigilance monitoring smartphone application to prevent road traffic accidents with audible smartphone alerts.

#### 4.3.2. Driver Performance Assessment

For most people driving is a habitual and easy activity as it tends to be second nature to perform driving manoeuvres due to daily repetition. However, driving is a complex decision-making process because of the dynamic nature of various components. Components such as the driver, the vehicle and environment, as well as the relationship between these entities should be a key focus in driver performance assessment [10]. Driving capabilities of a person depend on the age of the driver, driving experience, their physical, emotional and psychological/mental state, as well as environmental influences such as the weather or the time of day.

Four driving style classes were categorised and applied in a driving performance inference system, with the signature of multi-dimensional acceleration at the core of the solution, with the aim to allocate all possible driving events to one of these classes [13]. The proposed solution, performs classification in real-time, and proves to be cost-effective.

*EcoSmart* is a mobile application that addresses fuel consumption improvement based on driving style as part of a remedial energy consumption and pollution strategy [44]. The *EcoSmart* application uses the principles of driver behaviour assessment from the kinematic data (acceleration and speed), in order to cooperate the driver to correct and adopt behaviour to proceed towards the ideal driving behaviour for optimal fuel consumption and energy emissions.

#### 4.3.3. Riding Comfort Improvement

**Figure 2:**
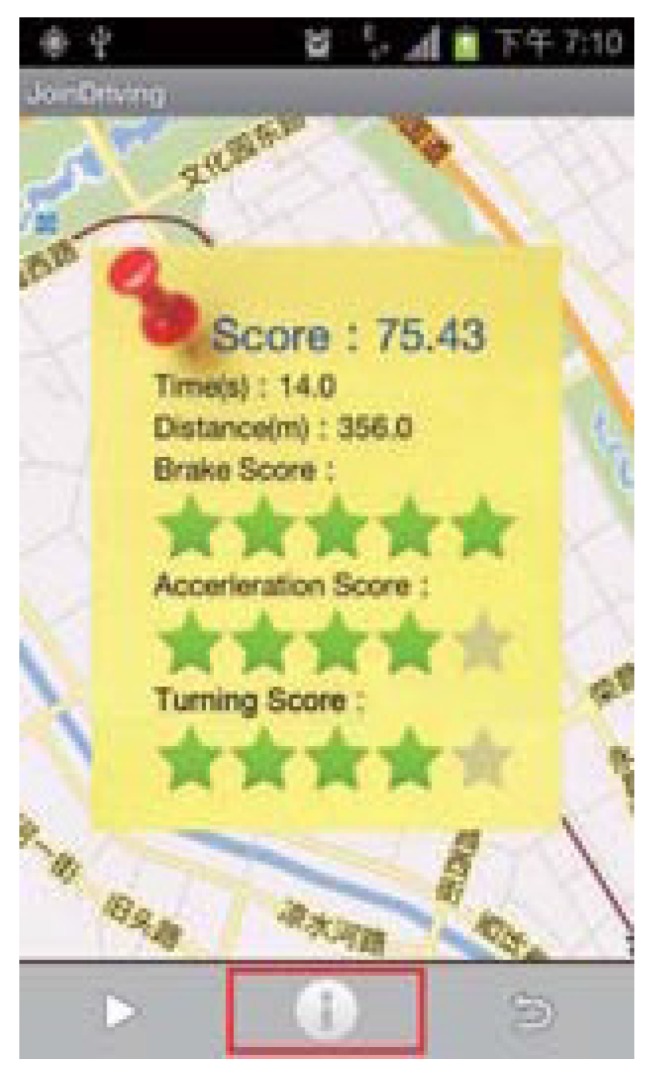
*JoinDriving*: Riding comfort related scores for an example driver [22].

A smartphone application named *JoinDriving* [22] addresses riding comfort levels with the application of a scoring technique to quantify the degree of aggressiveness, and indirectly also the degree of comfort experienced by passengers, as seen in Figure 2. Another example of an encouraging application that assists in improving one’s driving by means of a risk assessment scoring algorithm is *MobiDriveScore* [42]. Driver experience and competence contribute greatly to the safety of the driver. Expert drivers, for example, have the ability to effectively decelerate when approaching into a curve, and steer the vehicle in such a manner that the front wheels are subject to increased vertical load with more cornering force, which effectively improves steering movement and tire wear [8], and therefore improve driving ability will enrich riding comfort levels experienced by passengers. Drivers who follow smooth trajectories and systematically adjust acceleration levels in synchronization with the movement of the steering wheel are classified as safer drivers and this behaviour also improves ride comfort for the passengers as result [8].

### 4.4. Eco Driving

The concept of “eco driving” (economic driving) represents a driving culture where smarter and safer driving results in more fuel-efficient driving which contributes to environmental protection and pollution prevention. According to [8], the difference in fuel consumption (and thus gas emissions) between a normal driving style and an aggressive driving style is estimated to be above 40%, in favour of the normal driving style. Therefore, the promotion of eco driving will greatly contribute towards green projects and cost-effective driving due to improved fuel efficiency. The *EcoSmart* and *TutorDrive* collaborated project in [44] directly address the application of improved driving economy.

The properties of urban driving that have the greatest effect on emissions and fuel consumption are described [45]. Driving pattern parameters such as speed, acceleration and gear-changing behaviour were investigated, and the influence of these parameters analysed through regression analysis. The analysis was dependent on the environmental impact such as the route choice and was included in the study. It was found that speeding in itself does not influence emission considerably, and the attempt to lower speed limits for traffic safety reasons are not key problems from an environmental point of view. Instead, it was motivated that the main focus should fall on adapting environments, driving style and vehicles in a way that discourages heavy acceleration, power demand, and high engine revolutions. An eco-friendly and safe driving style can therefore be maintained with the avoidance of sudden accelerations, rapid braking, and cornering actions.

Real-time fuel consumption optimization is performed in many driver behaviour applications, and in [17] the driver is made aware of fuel burdensome actions by assessing driving behaviour with a smartphone-based system. Another solution based on Fuzzy logic, that makes use of a driver scoring approach, addresses fuel consumption using a smartphone [38]. The emergence of electric drive vehicles, like hybrid electric vehicles (HEVs), promises a considerable decrease in fuel consumption and greenhouse gas emissions [46]. However, this benefit offered by HEV technology will be greatly influenced by driving behaviour that deviates substantially between drivers. A personalised driving behaviour monitoring system for hybrid vehicles was introduced that addresses vehicle performance enhancement, to fill the vacant market for HEV fuel optimization [46].

### 4.5. Road and Vehicle Condition Monitoring

Condition monitoring of either the road or the vehicle is an important aspect of road safety, as deteriorating or damaged roads hold safety risks for travellers, possible vehicle wear and reduced driving comfort for travellers.

Monitoring the road surface condition in real-time enables effective and informed road maintenance planning and dispatch. A pattern recognition system on a smartphone was developed in [47] that detects road condition from accelerometer and Global Positioning System (GPS) readings. This was accomplished through spectral analysis of tri-axis acceleration signals to retrieve trustworthy road surface anomaly information. The accelerometer data and techniques used in this application are similar to those applied in classifying driving styles, and therefore it is concluded that road surface condition monitoring functionality can also be adopted in the applications of driving style and driver behaviour analysis, through driver-vehicle coupling techniques.

*Wolverine*, a non-intrusive method for traffic and road conditions detection was introduced in [48], using accelerometer, GPS and magnetometer sensor data. This application analyse braking events (regular braking can indicate traffic congestions) and bumps in the roads (characterise the type and condition of the road). The work of [38], indicated that the classification of a particular road’s overall integrity is not as simple as previously proposed solutions. But despite of this fact, the study proved successful in classifying potholes, bumps and surface type (rough, uneven, and smooth surfaces) using multi-axis accelerometer readings, while the road condition monitoring service provides feedback to the driver regarding unsafe road characteristics in order to improve road safety.

**Figure 3:**
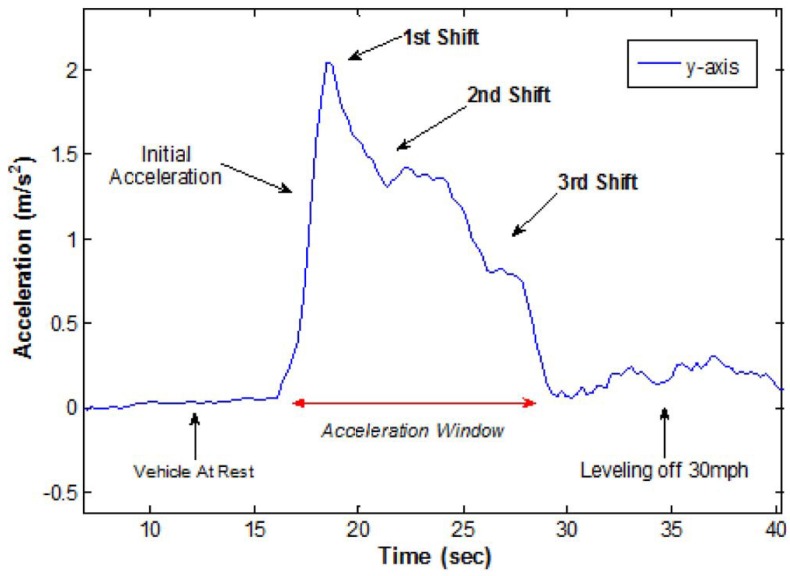
Engine gear shift analysis of a vehicle based on the y-axis accelerometer data [38].

The lack of knowledge of a properly functioning vehicle, *i.e*., a vehicle that is in a good or normal condition, is an area of concern for many drivers. A vehicle that is in a bad condition is a safety risk for the driver of the vehicle and other road users, because of the increased probability of, for instance, engine problems during acceleration and high-speed driving, and slipping of gears due to wear. A method investigated to monitor vehicle condition was presented by the authors of [38], where a mobile smartphone implementation was capable of recognizing gear shifts and irregularities in gear shifting patterns. In this study optimal gear shifting of manual transmission vehicles, for efficient fuel economy, was listed to be in the range of 2300 to 2500 rev/min. It was also mentioned that in the case of automatic transmission vehicles, the recognition of gear slippage can be useful information for early warning of possible gearbox depreciation, low transmission fluid, worn clutch discs, or a faulty shift solenoid. A graphical representation of the gear shifting results, obtained from this implementation, can be seen in Figure 3.

### 4.6. Fleet Management

The area of fleet management is rapidly growing, and includes a range of applications such as vehicle maintenance, vehicle telematics (tracking and diagnostics), speed monitoring, driver monitoring, fuel management and health and safety inspections [21]. Through effective fleet management, companies can minimise the risk vehicles and drivers are subject to, improve efficiency of their service and reduce overhead costs. Some of these advances are now also driving government legislation in various countries. *MIROAD* (Mobile-Sensor-Platform for Intelligent Recognition Of Aggressive Driving), is an inexpensive, accessible and intelligent system designed and implemented in [21], that satisfies certain fleet management requirements without mounting expensive camera and surveillance monitoring devices in a vehicle, but rather uses driver behaviour and driving style analysis techniques and a mobile sensor platform in order to detect aggressive driving. Other fleet management systems that utilise two-axis accelerometer and GPS readings, (for instance the application proposed by [13], and the practically tested intelligent fleet management system in [49]), are normally implemented on a web server with a web interface, where organizations can monitor and optimise their fleet in an easily accessible and effective manner. These fleet management systems focus on the importance of feedback and history tracking.

The behaviour and integrity of a driver is essential in fleet management and fleet optimisation, as the driver takes responsibility for the fleet vehicle, which is a costly company asset, as well as the vehicle’s load. Therefore, driver performance scoring practices can prove valuable in assessing the risk of an individual driver. Such applications include *SenseFleet* [43], which identifies risky manoeuvres performed by the driver using a Fuzzy Inference System (FIS). Evaluation experiments on *SenseFleet* have resulted in accurate identification of risky driving manoeuvres based on acceleration, braking and cornering actions, and also provided representative scores for a large number of test cases. Another functionality of *SenseFleet* provides road network and traffic information feedback, which aids in the tasks of fleet management and route optimization.

Currently telematics is a leading technology in fleet management, where data acquisition is performed by “black box” tracking devices or on-board vehicle diagnostic devices. In [50], the prediction of future behaviour using real-time and historical data from telematics devices, enables the modelling of road-vehicle interaction which provides condition based maintenance information and other fleet management information specific to the driver. Scoring techniques are applied, and in this instance a hazard rate (HR) score is generated for each trip. The trip information with the accompanying HRs are used to model complex road-vehicle interaction where degradation of the suspension-damper system could be identified, and an algorithm could estimate hazard rates for different road types to enable better and safer route planning.

### 4.7. Accident Detection

Real-time acceleration and jerk variance evaluation for individual vehicles are often used to detect anomalous behaviour in risk assessment [51]. Accident detection is performed mainly based on methods that monitor traffic density as described in [52], but real-time driver behaviour and vehicle movement analysis systems can also prove relevant and immediate. Certain early-accident detection applications include immediate dispatch of emergency services and road-side assistance services directly upon accident detection [53]. This means that emergency assistance services can be aware of accidents, and the severity thereof, before or without the incident being reported. Accidents can be detected based on impact readings based on longitudinal and transversal acceleration measurements. In [26], an innovative system is presented to integrate accident detection with driver behaviour and driver style classification systems. This is done based on critical jerk evaluation, which enables safety critical braking event identification and accident occurrence recognition from real-time driving data, due to the high correlation between them. The jerk method that was performed had a success rate of 1.6 times greater than the longitudinal acceleration methods.

### 4.8. Insurance Applications

The insurance industry, especially the motor insurance sector, generally calculate their premiums based on statistical data through the evaluation of factors that are believed to impact expected cost of future claims. These factors include among others, the type of vehicle, the value and characteristics of the vehicle, as well as the profile of the driver (age, gender, marital status, driver experience, *etc*.), which do not always fairly represent each individual driver’s propensity to risk. However, driving behaviour analysis can assist to provide a more accurate representation of an individual. The results in [43] indicated that dangerous driving events are accurately detected and provided a representative score for each individual driver.

GPS vehicle tracking through vehicle-fixed “black box” units is promoted by insurers, as the information retrieved from these units can be used by the insurance companies to better estimate risk. The *DriveDiagnostics* system monitors on-road driver behaviour, calculates risk and provides relevant feedback using data from vehicle-fixed data recorders [54]. Methods based on vehicle-fixed sensor data acquisition are unappealing to some people who find the idea of constant monitoring invasive.

**Figure 4:**
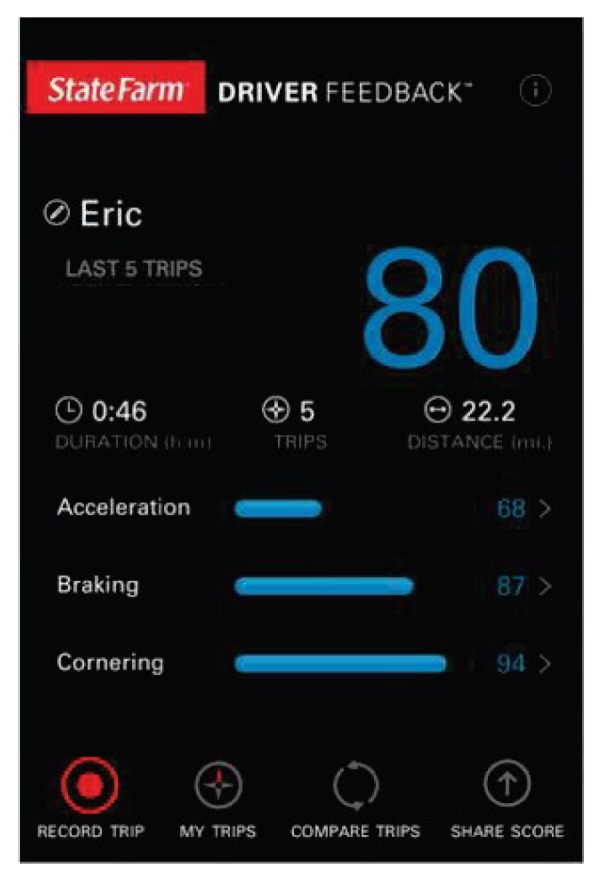
Smartphone interface for a behaviour based insurance scoring application [55].

Another approach recently exploit is behaviour-based insurance combined with usage based insurance (UBI), which makes use of driver behaviour patterns and driving style classification to improve assessment of driver risk and insured risk. An example of such an approach that moves away from vehicle-fixed systems is proposed in [55] (Figure 4), where new degrees of freedom are introduced with a smartphone as sensing platform. The smartphone based system also functions as a driver assistance or support tool [27]. This type of system can afford the driver the chance to reduce his risk profile, and by doing so, qualify for a reduced insurance premium. Examples of such a system are the Pay-As-You-Drive (PAYD) and UBI schemes [56]. In PAYD insurance plans the initial premium is based on the driver’s risk profile. The premium is then recalculated after a predefined evaluation period based on accelerometer, GPS and on-board diagnostics (OBD) data. The down side of this system is that the customer does not have an indication of his progress because the device data is not accessible by the insured party. *MobiDriveScore* was therefore developed in order to enable the customer to assess his personal driving pattern and score using a smartphone [42].

### 4.9. Hijacking Detection

Anti-hijacking precautionary methods are currently approached in a manner of real-time panic alerts, that is normally implemented as a hardware switch that notifies a control room or activate an immobiliser on the vehicle. This type of action cannot be classified as driver behaviour, as it is a sporadic trigger in case of emergency. Although driver style analysis is used for driver safety in [21], anti-hijacking is also discussed in this work, with mentions that a software application might not be able to prevent a driver from being hijacked.

A valuable application using driver behaviour and driving style analysis techniques would be able to detect hijack occurrence shortly after the hijacking took place, by recognising different driving patterns and notifying authorities of suspicious activity and the possibility of a hijacking having just occurred. This application can assess the driver’s behaviour and based on differences identified between the different driving styles the application might be able to infer that another driver is driving the vehicle. An innovative method for identification of individual drivers is proposed and implemented in [8], using features such as frequency of indicator use when turning, braking frequency, favourable gear, gear shift frequency and average speed. The need for such a system differs between countries, but at the moment such an high-jacking detection application, and research toward its success, is in demand in developing countries where hijacking and vehicle theft are notably higher compared to first world countries. Currently the literature does not provide substantial theft detection related studies applying driver behaviour information and features.

### 4.10. Intelligent Vehicles Systems and Autonomous Vehicles

**Figure 5:**
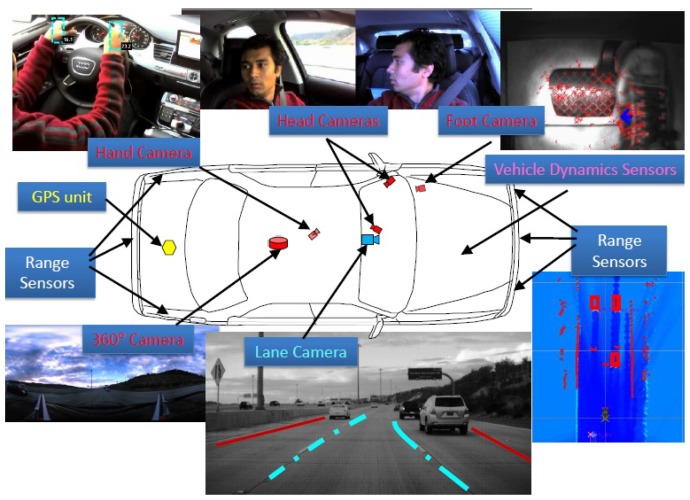
An example of on an Intelligent Vehicle used for data collection [57].

Intelligent vehicle technologies consist of electronic, electromechanical and electromagnetic devices that are controlled by software-based control systems. These intelligent vehicle technologies are applied to vehicle safety systems and self-evaluated feedback systems [14,29]. State of the art CAN vehicle bus standards using OBD data and telematics sensors collect as much information from the current vehicle’s movement and surrounding vehicles’ movement as possible, and estimate real-time feedback to provide solutions and suggestions to the driver. Intelligent vehicles are usually equipped with multiple sensors to enable various processing with ample and representative data collection from various components of the vehicle and the driver behaviour. An example of an intelligent vehicle equipped with multiple sensors is illustrated in Figure 5, which contains in-vehicle camera sensors, vehicle dynamics sensors, range sensors and a GPS unit [57].

Information assessed by intelligent vehicle systems (IVS) might include vehicle orientation estimation using GPS data, relationships between the yaw angle and the steering wheel angle using inertial measurement units (IMU) and lane changing trajectories [58]. Driving experience is improved in [59], through the automated calculation of physiological features while driving. This information is obtained by continuously recording electrocardiogram, electromyogram, skin conduction and respiration, and used in IVS to provide feedback to the driver suggesting behaviour to improve relief and relaxation. In order to prevent risk, IVS can divert an incoming cell phone call to the mail box if it is determined that the mental or emotional load of the driver is above average, for instance during high stress, and address the needs of a driver who is more prone to distraction in low stress situations. Many interesting capabilities exist for features of IVS, but driver behaviour modelling and driver style classification play an important role in autonomous vehicle research, which also forms part of vehicle intelligence.

In order for an autonomous vehicle to travel safely, the autonomous vehicle should interact with the environment as well as with other vehicles. It is crucial that the autonomous vehicle should interact with human driven vehicles, and therefore the future driver behaviour prediction or driving style information of the approaching human driven vehicle can prove valuable for movement and steering decisions performed by the autonomous vehicle in order to avoid traffic accidents [60]. In [39], a hybrid-state system is developed and tested by decision-behaviour coupling of different systems, in this case, coupling of the driver and the autonomous vehicle.

Inter vehicle communication is essential for IVS enhancement. As a result, vehicular ad hoc networks (VANETs) originated as an application of mobile ad hoc networks (MANETs), which allow vehicles to communicate with each other in short range proximity. This technology already addresses road safety through feedback to the driver of risky surrounding activities, applying driver behaviour analysis applications using VANET technology, and is implemented in [14]. Autonomous vehicles and intelligent vehicle systems make use of vehicle following models for navigation as well as driver safety applications through dangerous following distance detection [61]. A driver assistance application which evaluates vehicle following models and patterns recorded for a driver, to provide functionality such as adaptive cruise control and forward collision warning, is introduced in [62]. IVS show promising development potential for state of the art driver behaviour analysis applications.

**Figure 6:**
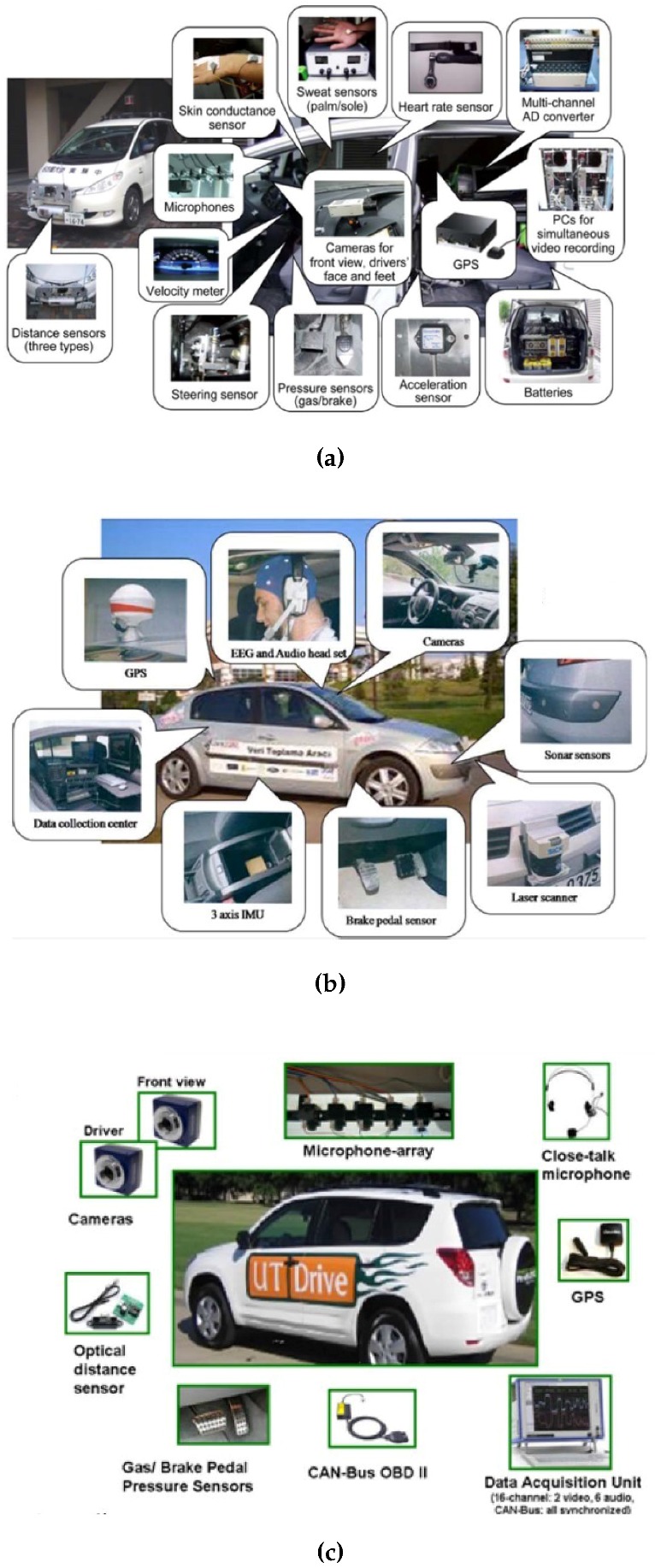
Sensors used for data collection in intelligent and autonomous vehicle studies [41]. (**a**) NUDrive; (**b**) Uyanik; (**c**) UTDrive.

Amalgamated results and findings obtained by collaborating researchers from three different regions (Asia, America, and Europe) in the area of driver behaviour analysis, were merged into one product to serve as a global solution [41]. Intelligent vehicle systems were used for data collection in their research, and an illustration of the *NU-Drive*, *Uyanik* [63], and *UTDrive* [64] vehicles and the equipment(distance sensors, skin conductance sensors, heart rate sensors, cameras, pressure sensors, GPS, steering sensors, EEG sensors, sonar sensors, laser sensors and IMU sensors) fitted to these vehicles are illustrated in Figure 6.

## 5. Underlying Algorithms

In this section the popular algorithms (AI algorithms, ML algorithms and statistical algorithms), and their roots and applications in driver behaviour and driving style studies are summarised. Significant results are provided when applicable, with the intention to identify suitable algorithms to pursue in unique driver identification problems. It was noticed that there is a relationship between the algorithm used and the application it serves. Artificial Neural Networks (ANNs) find its application in drowsiness detection [32], driver distraction detection [28] and steering behaviour prediction [65] and is mainly based on computer vision techniques. A frequency domain solution to address temporal input complications, is performed using a Fast Fourier Transform (FFT) in a drowsiness detection problem [32]. Clustering techniques were used for driving style distinction and labelling in [43] and k-means clustering, in specific, for individual driver identification [51] and road condition monitoring [48]. State Machine approaches are performed in driver manoeuvre recognition [66] and driver decision making modelling [67] in the form of a Finite State Machine (FSM), and in autonomous vehicle studies [68] using a Hybrid State Machine (HSM) form. Fuzzy Logic implementations include driver fatigue and distraction identification applications in [28], scoring applications in [43,56] and driving style recognition applications in [13,17,20]. Hidden Markov Models (HMM) are primarily applied in driving behaviour estimation [60,68], driving manoeuvre recognition [10,69,70], driving performance assessment [70] and driver distraction detection [41] studies. A Bayesian technique is applied in a driver behaviour detection application to address the problem of incomplete data in [14]. Decision Trees are utilized in [37] for result confidence estimation in an information fusion application for drowsiness detection. Gaussian Mixture Models (GMMs) was investigated in driver distraction detection [41], driving manoeuvre detection [71] and road condition monitoring [58] applications. Dynamic Time Warping (DTW) is applied in driver risk profile classification [72] and driver assistance or safety awareness [21] applications. A Kalman filter was used in the process to predict and model human behaviour in [73]. Support Vector Machines (SVM) are used for driving event recognition [12,74] systems, as well as in drowsiness detection [75] and vehicle state estimation [48] systems. Genetic Algorithms are applied in the calibration process of car following models as can be seen in [61]. Promising algorithms to further investigate in future research include Fuzzy Logic based techniques, HMM implementation and SVM based applications.

### 5.1. Artificial Neural Networks

An ANN is an intelligence system that mimics the biological interactions of the nervous system (the brain) and consist of various extremely interconnected processing elements, that solve problems with collective processing [28]. In an ANN, signals are transmitted through the connections between nodes of the network (neurons), each assigned with a characteristic and adjustable weight. The adjustable weights enables the network to be trained and to be optimised through a learning algorithm. In a standard ANN this weight is the multiplier with which the input is adjusted to obtain the output value. However, different types of ANNs exist to address problems in various fields. An activation function is used to define the output of the node that is fed further in the network.

ANNs are categorised as a machine learning algorithm because the network approximates activation functions and improves its approximations using a learning algorithm. It finds application in driver behaviour solutions and in this survey an investigation is performed to identify the reasons for ANN use, the significance of ANNs, and the results obtained by using the ANN as learning model in these applications. Human behaviour modelling is a suitable field of application for an ANN as human behaviour tend to be nonlinear and stochastic. According to [76], complications arise in certain applications (especially driver assistance applications) due to the difficulty to estimate the drivers’ physical and mental state, as well as the adequacy of the information acquired from these models.

Oppositely, results with fewer complications and high accuracy were obtained in other studies. One example hereof is the computer vision application performed in [32], for drowsiness detection where the percentage of eye closure (PERCLOS) was used as one input to an ANN in order to estimate the state of driver drowsiness. Driving information used as more inputs to the multilayer perceptron (algorithm applied to binary classification) ANN, included the lateral position, the steering wheel angle and heading error retrieved from the CAN bus. The study conducted in [32] further claims a drowsiness detection rate of 70% using single indicators and with the coupling of multiple indicators achieved a drowsiness detection rate of up to 94%. These results were obtained with a naturalistic driver simulator using driver video surveillance and driving signals, but complications will be introduced with actual driving data and situations.

A Feed-Forward Neural Network (FFNN) consist of a layered structure, and are considered static networks as they have no feedback operations and, therefore, the information is fed from one node to the next down the layered structure without delay and the output are solely dependent on the input. Apart from the static FFNNs, dynamic NNs exist which comprise of feedback elements and is used for prediction purposes.

In the driver distraction detection application performed in [28], both static and dynamic ANNs were implemented using vehicle dynamic data such as speed (m/s), time to collision and lane crossing (s), positions of the accelerator and brake pedals (%), steering wheel angle (deg), and lateral position (m). Dynamic ANNs require a back-propagation approach to train the network introducing more expenses to the solution as opposed to the static ANNs. An example of such a system can be found in [65], where dynamic ANNs were used to predict the steering behaviour of a driver based on road curvature and the speed and acceleration of the vehicle. Real driving data were used in the pre-mentioned study, and it was concluded that acceleration and velocity measures did not improve the steering behaviour prediction accuracy in the solution.

Another variation of an ANN application can be found in [61], where a fuzzy rule-based ANN model was used to obtain driver-specific driving constraints from the trajectory data of the vehicle. In this method the ANN was trained using an ML method known as reinforcement learning, in order to enable the system to imitate the driving behaviour of an individual. Uniformity between different drivers’ behaviour was also investigated in [61], and it was found that heterogeneities exist between similar driving actions.

Optimizing machine learning problems applying particle swarm optimization (PSO) is achieved through a classifier ensemble pruning method and it was found that the performance of an ensemble of classifiers is better than a single classifier with an ANN identified as a suitable base classifier [77].

The estimation of prediction intervals (PIs) is an extensive limitation in the application of ANNs to real-life problems. A recent solution that addressed this limitation in the operational streamflow forecasting industry, made use of a Lower Upper Bound Estimation (LUBE) method which outperforms classic PIs construction techniques such as the delta method, Bayesian methods, and bootstrapping on both synthetic experiments as well as real world regression problems [78]. Consideration of this technique could prove relevant in driver behaviour model construction.

Due to a conventional ANN resulting in poor rainfall forecasts in initial experiments, a hybrid model integrating ANNs and support vector regression (SVR) was developed for daily rainfall prediction [79]. In the modelling process, singular spectrum analysis (SSA) was first adopted to decompose the raw rainfall data followed by fuzzy C-means clustering to split the training set into subsets associated with the intensity of rainfall. The joint effect of a hybrid ANN-SVR model and the SSA filtering technique considerably improved the accuracy of daily rainfall forecasting. An improvement in daily flow prediction was also identified by coupling three data-preprocessing techniques, moving average (MA), SSA, and wavelet multi-resolution analysis (WMRA), with ANNs [80].

### 5.2. Fast Fourier Transform

Frequency domain approaches have been investigated in the drowsiness detection study performed in [32], due to the absence of the mean and standard deviation measures of the steering wheel angle and heading error in the time domain. It is assumed that drivers who are conscious will perform gradual steering wheel corrections, where a sleepy or fatigue driver will perform impulsive and sudden steering wheel movements. The spectral energy distribution is assessed in the inference of drowsiness detection with an FFT in [32] and it is was hypothesized that concentrated spectral density indicated the conscious state and scattered and stretched spectral density indicated a state of drowsiness.

### 5.3. State Machines

A state machine is a mathematical abstraction that is applied to design algorithms. The states of the system specify which state to switch to in different situations. A deterministic FSM is an FSM for which only one transition exists for any valid input, as opposed to a non-deterministic FSM, which is an FSM where a given input from a particular state can lead to more than one different states. Examples of applications for finite state machines and hybrid state machines are given in this section.

#### 5.3.1. Finite State Machines

**Figure 7:**
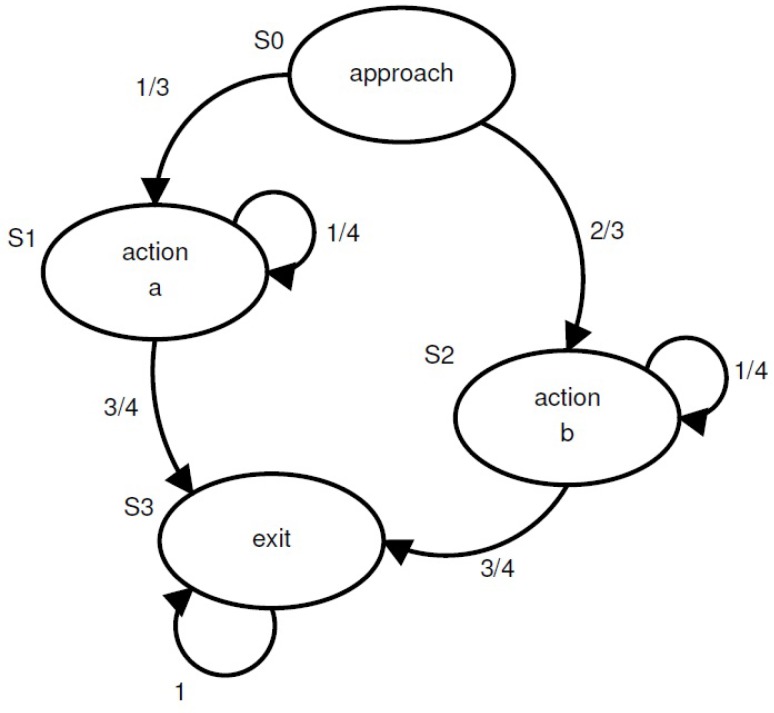
The Finite State Machine (FSM) model and transition probabilities for driver decisions at an intersection, where an approaching vehicle has two possible choices [67].

In the driver manoeuvre recognition approach in [66], a probabilistic FSM is used to completely capture the possible sequences of the core elements that represents a driving manoeuvre. The feasibility of this approach is justified through real driving data and recognition of turn manoeuvres with low computational complexity. In the Intelligent Transportation Systems (ITS) study conducted in [67], driver decision-making is modelled stochastically in order to develop a human driver model. A FSM design guideline is proposed for a driver decision-making model, to maximise state observability and resolution while limiting the size of the state machine to realistic proportions. A graphical depiction of the state transition topology of the FSM design, proposed for a probabilistic decision making model at an intersection, is given in Figure 7. The driver has to make a decision for instance to turn at the intersection, to stop at the intersection or to continue the original trajectory of the vehicle. This transition model can be adapted according to the needs of a particular application.

#### 5.3.2. Hybrid State Machines

A framework for driver behaviour estimation at intersections is proposed in [68] in which an HSM framework is designed, comprising of a discrete state system and continuous state system, to address autonomous driving and vehicle safety requirements. The driver behaviour (continuous state system) and vehicle dynamics (discrete state system) is modelled as a HSM in order to ensure vehicle-driver coupling. This particular system makes use of a HMM to perform the driver behaviour estimation. Driver state estimation is also performed in [60], which relies on rule based estimation due to the fact that the vehicle trajectory will follow the action performed by the driver.

### 5.4. Graphical Methods

A graphical model also known as a probabilistic graphical model is a model for which the conditional dependence structure between random variables is expressed. Graphical models find application in probability theory, Bayesian statistics and machine learning. According to [70], graphical methods can also be described as directed acyclic graphs (DAGs) or probabilistic inference networks (PINs) which function is to provide computational efficient solutions to time domain analysis and modelling. The popular graphical methods applied in driver behaviour systems are investigated and it is clear that HMM approaches are popular in the literature.

#### 5.4.1. Hidden Markov Models

According to [81], a HMM is a double stochastic process and is capable of learning structure intelligence from a set of observation sequences. A HMM is therefore suitable for the problem of driver behaviour estimation, which requires a model that can represent the relationship between continuous and discrete state observations for instance the relationship between the driver’s actions (changes in orientation) and the actual movement of the vehicle. According to [60], a HMM can satisfy this requirement due to its architecture by completely specifying these dual states, in the instance where actual driving data is used for training the HMM.

In [69], HMMs are used in the final phase of the driving manoeuvre recognition and driver fault diagnosis application performed in an urban road scenario. Classification cannot be performed with linear approaches and therefore an ANN is implemented, with the capabilities to recognise and classify driving manoeuvres if the ANN is trained prior to classification. This system is capable of vehicle’s manoeuvre sequence identification, test appropriateness for a certain urban road driving event, differentiate between an inattentive, or drunk driver and a normal driver. Simulator data were acquired in the tests performed in this study, and in future research conducted by [69] real driving data will be used. The labels classified by the ANN are used to train the HMM.

Another driving manoeuvre and driver’s performance evaluation application was conduced in [70], this system however used real-time data acquisition in contrast to the prior solution which made use of driving simulator data. In [70] video signals of the traffic, the driver’s head and the driver’s viewpoint, were used in conjunction with signals obtained from the vehicle (vehicle brake, gear, steering wheel angle and speed signals). In this application the predictive power of this solution is 1 s, which means that the HMM can predict the manoeuvre accurately one second prior to the actual action is performed. This is an important area of interest in autonomous vehicle studies. A top-to-bottom (TtB) HMM approach was implemented in [71], by identifying a major manoeuvre and then the discovered manoeuvre is parsed into finer more characteristic manoeuvres. A hierarchical framework is proposed and it is believed that the framework that combines the bottom-to-top (BtT) and TtB approaches, will produce significant recognition improvements.

Other applications of HMMs include the addressing of the temporal aspect of multi-level classification to adapt varying driving styles and road conditions, to improve detection rates [32], and driver behaviour estimation, in which continuous vehicle observations are modelled as GMMs for the learning and evaluation of HMMs [68]. Improving performance by introducing a HMM as an extent to previous work performed by the authors in [41], lead to a detection rate ranging from 80% to 90% for driver distraction detection and driver behaviour estimation, in which continuous vehicle observations are modelled as GMMs for the learning and evaluation of HMMs [68]. A simple and reliable model for driving event recognition applying HMMs was performed in [10], using longitudinal- and lateral acceleration data.

#### 5.4.2. Bayesian Networks

Bayesian Networks address the limitation of incomplete data in cases where ANNs, Fuzzy logic and techniques such as the Kalman filter will not provide enough expressive capabilities. In [14], driving style classification between four driving styles (normal, drunk, reckless, and fatigue) is performed in real time by introducing a probabilistic model based on dynamic Bayesian networks (DBNs). Contextual information regarding the driver, the vehicle, and the environment are fused together. The dynamic behaviour model captures the static and the temporal aspects of the driver’s behaviour and led to robust and accurate behaviour detection.

### 5.5. k-Nearest Neighbour Classifiers

The k-Nearest Neighbours (k-NN) algorithm is a well-known nonparametric classifier which is based on simple and effective supervised learning and classification techniques. Supervised locally linear embedding (SLLE) proved to be a powerful feature extraction method in the feature extraction for plant leaf recognition [82]. A recognition rate of greater than 90% was achieved on five kind of plants using an input vector for the k-NN algorithm. The extraction of driving event features are necessary and the SLLE method should be investigated.

### 5.6. Decision Trees

A decision tree is a decision support mechanism that uses a tree-structured graphical model of decisions and the possible consequences of them, this may include resource cost and complexity of a decision. Decision trees can be used to classify or choose multi-source data collected from a variety of sensors. Decision Trees provide a thorough structure representation of data selections and outcomes choosing those selections.

In the study performed in [37], a decision tree implementation was responsible for the reasoning stage to judge the driver drowsiness states. The threshold values need to be adapted appropriately for different drivers due to differences in driver habits and driver behaviour.

### 5.7. Fuzzy Logic

Another area of great interest in driver behaviour prediction and recognition is the usage of the fuzzy logic (FL) principle. Fuzzy logic represents logic as a degree of truth or in some cases many valued logic is imposed instead of binary logic known as true or false. Fuzzy sets were introduced in 1965 as a class of objects with a continuum of grades of membership [83]. These sets are characterised by a membership function which allocates a grade of membership to each object ranging between zero and one, this is to address precision in logic when addressing human reasoning [66].

A fuzzy interface was used in [31] to detect driver fatigue, and in [28] an adaptive neurofuzzy inference system (ANFIS) was used to detect driver distraction. The reason for the implementation of an FL system (FLS) in [28], is due to the ability of a FLS to simultaneously interpret numerical data and linguistic knowledge and its ability to map nonlinear input data to a scalar feature vector output. The membership functions applied in FLS are normally curves with a triangular shape or a Gaussian shape. Defuzzification is the estimation of the output fuzzy sets of the FLS into a single number. The nonlinearity and structured knowledge representation are the primary advantages of ANFIS over classical linear approaches in adaptive filtering and inference systems.

**Figure 8:**
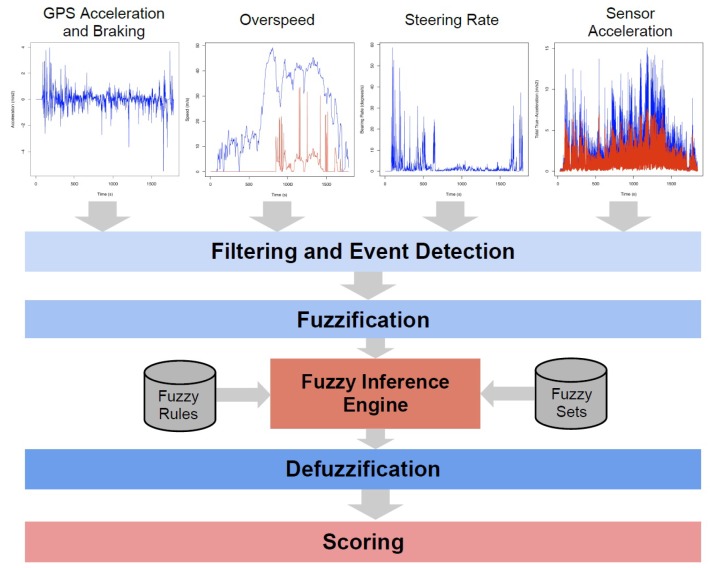
Fuzzy Inference System [56].

An area in driver behaviour and driver style analysis where FL finds valuable application is in driver performance or risk scoring applications. An example of such a system is presented in [43], where a FLS is used to compute a score for different drivers. It was found that this implementation accurately detected risky driving events and a representative score for each individual driver through a FLS. The FLS design of the fuzzy-logic based scoring mechanism applied in [56], can be found in Figure 8.

**Figure 9:**
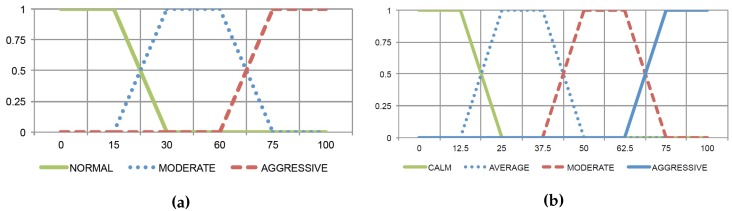
Deviation in the output variable categorization in driver scoring applications. (**a**) Output Fuzzy Set [84]; (**b**) Output Fuzzy Set [56].

Smartphone sensory data was used in this study that included accelerometer and GPS data, the sensing data was filtered after which event detection was performed. The input data was fuzzyfied thereafter and fuzzy rules were applied to infer conclusions. The defuzzification produced a score (between 0 and 100) and was used to provide each driver with personal feedback. In [84], the output variable represented three categories (Normal, Moderate and Aggressive) whereas in a similar study by the same authors conducted afterwards, [56], the output variable was extended to four categories (Calm, Average, Moderate and Aggressive). This was done to address increased complexity in the profiling algorithm as an increased number of output categories led to a better representational score. The output fuzzy set of both these examples can be seen in Figure 9.

Fuzzy inference techniques were further applied in driving style recognition studies, namely the study conducted in [20] which yielded a 68% classification confidence using vehicle dynamic data, the study performed in [13] where 2-axis accelerometer and speed readings were integrated, and [17] which made use of the three-axis accelerometer data to determine not only driving style but vehicle body posture as well.

### 5.8. Clustering

**Figure 10:**
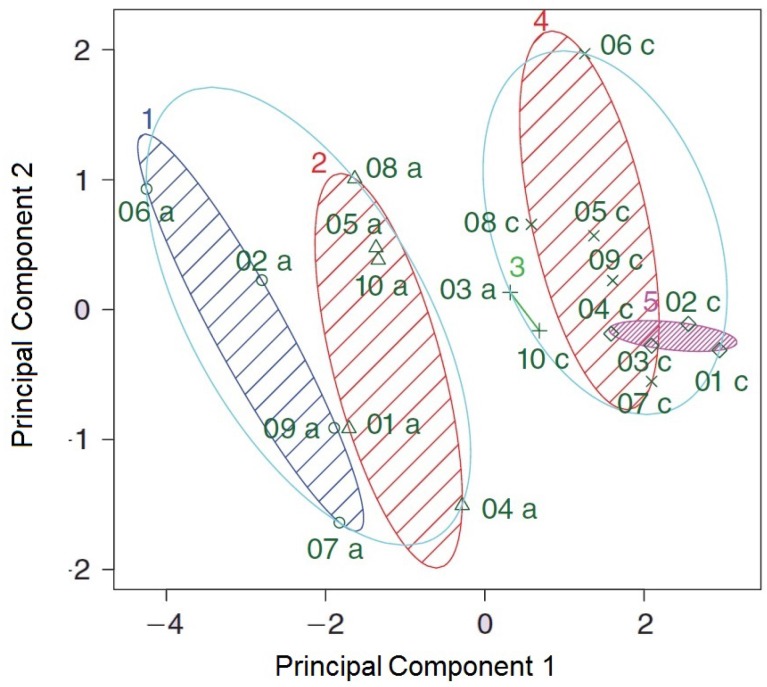
Driving style Lap clustering [43].

Clustering is a form of unsupervised learning where a dataset requires labelling and the intuition is by classifying items with the same label if they are clustered together or concentrated in a similar region. An illustration hereof in a driver behaviour scoring implementation in [43] is depicted in Figure 10. In this study the *SenseFleet* application was used as data acquisition sensor and drivers completed laps on a similar marked road portraying different behaviour while driving. The distinction between a calm and aggressive lap could be made through clustering techniques, where a calm lap will have a lower number of events and the aggressive lap a much higher number of events. The platform output was compared with the drivers’ subjective scores as measured by *SenseFleet*, and the results were categorized into five distinctive classes, these five clusters are labelled in the illustration shown in Figure 10. It can be seen that the distance between the calm and the aggressive clusters illustrates that *SenseFleet* enables for a clear distinction to be made between both driving styles. They observed that the separation between the two driving styles was at a score of 47.75 after using clustering to separate the labels effectively.

#### 5.8.1. Gaussian Mixture Models

A mixture model is used in order to make statistical inferences about the properties of a sub-populations in the case where observations are made only on the pooled population, without sub-population identity information. GMMs are the mixture of Gaussian probability distribution functions (PDFs). In [68], a GMM was applied of the form of

(1) $$b_{j}\left(o\right)=\sum_{k=1}^{M}c_{jk}N\left(o,\mu_{jk},U_{jk}\right),1\leq j\leq N$$

to represent such PDFs of continuous vehicle data, where *c* represents the mixture coefficient for the *k*-th mixture in the *j*-th state. *N* represents the pdf of a normal distribution with mean *μ* and covariance *U* measured at observation *o*. The mixture coefficient *c* satisfies the constraints in

(2) $$\sum_{k=1}^{M}c_{jk}=c_{jk}>1,1\leq j\leq N,1\leq k\leq M$$

such that the pdf is normalised to

(3) $$\int_{-\infty }^{\infty }b_{j}\left(o\right)d0=1,1\leq j\leq N$$

GMMs are used to perform driver distraction detection as a binary decision problem [41] and driver manoeuvre detection (optimal using a GMM with 64 mixtures) [71]. Road condition monitoring applications also apply GMM to classify road surface as either even or uneven using the yaw angle of the vehicle to measure the road surface [58].

#### 5.8.2. k-Means Clustering

Classification of unique individual drivers is an area of interest in this study and in [51] an innovative idea for the classification of individual drivers is proposed and experiments performed on different clustering methods, with the k-means outperforming the rest. K-means clustering is also performed in [48] for traffic and road condition estimation. For bump detection in this application the K-means clustering algorithm is performed on the extracted features and the result is fed to the manual labelling stage, after which it is used to train a Support Vector Machine (SVM).

### 5.9. Dynamic Time Warping

In time series analysis, DTW is an algorithm for measuring similarity between two temporal sequences which may vary in time or speed. Dynamic Time Warping is a vital tool to utilise in driver behaviour studies. Examples of DTW implementation can be found in [72] that follows the following system flow in order to predict driver risk profile knowledge. The driving data is fed to a smoothing algorithm after which manoeuvre detection is performed. This is followed by a warping algorithm (DTW), and a Bayesian classifier performs the classification of a risky or safe driver. A driver safety awareness application is implemented in [21], that performs DTW to detect, recognise and record these safety critical actions. A distance function is defined, and the sum of the distances along the warping path *p* describes the total cost $c_{p}$ of the alignment path. The cost function used in [21] is

(4) $$c_{p}\left(X,Y\right):=\sum_{k=1}^{K}c\left(x_{mk},y_{nk}\right)$$

### 5.10. Kalman Filtering

The Kalman filter is used in driver behaviour studies and therefore a quick introduction to the concept will be provided in this section. The Kalman filter is defined by

(5) $${\hat{X}}_{k}={\hat{X}}_{k}^{*}+K_{k}\left(Y_{k}-h\left(X_{k}^{*},t\right)\right)$$

The optimal linear estimate ${\hat{X}}_{k}$ of the state vector is retrieved in the case where the Kalman gain matrix $K_{k}$ is chosen correctly.

In [71], a Kalman filter is used to estimate the vehicle output due to driver inputs and the prediction errors are fed into the HMMs. Kalman filters are similarly applied in the driver behaviour applications described in [14,56,58,69] where a Kalman filter is used to classify input parameters. According to [73], human behaviour is easily described as a set of dynamic models (Kalman filters) sequenced together by a Markov chain.

### 5.11. Support Vector Machines

In supervised learning applications the labels to which inputs should be matched are known and therefore the machine is aware of the classification that it needs to reproduce. A system that assigns labels to unseen instances is known as a classifier. SVMs are rather insensitive to the curse of dimensionality and are efficient enough to handle large scale problems in both sample and variable space, with the fundamental functionality to map data to a higher dimensional space via a kernel function [28].

In [12], a SVM method is used to recognise driving events and the results of applying a Gaussian radial basis function (RBF) kernel as opposed to K-Mean clustering are evaluated. The optimal recognition rate achieved was 60%, and it was observed that acceleration event data alone did not meet expectations. Better results were obtained using supervised learning as opposed to unsupervised learning, with the conclusion that shortened datasets outperformed the full dataset probably because of the RBF kernel. The study conducted in [74] also implemented a RBF kernel using the *UTDrive* instrumented vehicle as the sensor platform. The feature vector applied to perform the manoeuvre recognition task consisted of 28 signals, of which, 5 signals are obtained from the CAN-bus and 23 signals are obtained from a portable device (accelerometer signals, vehicle speed signals and bearing signals retrieved from the GPS sensor). Driver manoeuvre detection is performed using SVMs in [74], and in order to perform multi-class classification with a SVM, different approaches exist and in this instance a SVM was used with a Gaussian RBF kernel.

In [28], the authors proposed a nonintrusive method for real-time detection of visual distraction, using vehicle dynamics data and without using the eye-tracker data as inputs to classifiers. Their results proved that using SVMs outperformed the other machine learning techniques, providing the highest classification rate for most of the test cases. These results are especially important as it is applied to partially autonomous driving assistance systems (PADAs) in order to guarantee and ensure safety. SVM techniques have been applied in driving sleepiness detection algorithms with high robustness and practicability, some of these applications are listed in [75].

#### 5.11.1. Radial Basis Function

When training a SVM, the application of a kernel and specifically a RBF. The RBF is used in order to map samples of low dimensionality into higher dimensional space. The RBF used in [75] is given by

(6) $$K\left(x_{i},x_{j}\right)=e^{-\gamma {\left|x_{i}-x_{j}\right|}^{2}}$$

In this function, $i_{x}$ and $j_{x}$ are two data points, *C* and γ are the two unknown parameters for the RBF kernel. In [12], the power intervals for the unknown parameters was found as $C=[2^{-15};2^{15}]$ and $\gamma =[2^{-15};2^{15}]$.

### 5.12. Genetic Algorithms and Reinforcement Learning

In the field of AI, a genetic algorithm (GA) is a search heuristic that simulates the process of natural selection. Genetic Algorithms find its application in car-following models, in which two models namely Intelligent Driver Model (IDM) and Velocity Difference Model (VDM) were calibrated using genetic algorithms [61]. Swarm intelligence was further investigated in this study through reinforcement learning applied to network route choice analysis and real time traffic signal control.

## 6. Conclusions

Road safety problems are a major area of concern in the transport industry especially in developing countries. Driving assistance applications are actively investigated to address these safety issues, with human behaviour ascribed as one of the main causes and accelerators of road safety problems, which confirms why driver behaviour is currently receiving extensive research focus. In order to propose a solution to further address road safety and other commercial problems through driver behaviour analysis, driver behaviour is extended into different driving styles, which each represents different driver behaviour aspects of the same driving scenario. The driving styles have different physical, psychological and incidental measurable aspects. In order to attempt unique driver identification using driver behaviour analysis, a model needs to be defined that will be representative of different aspects of driving and therefore the evaluation of four driving styles is proposed as opposed to the typical binary classifications (aggressive and safe driving styles). The driving styles proposed (aggressive driving style, inattentive driving style, drunk driving style and normal driving style) are compared and justified in this report. It was also found that offline driver behaviour metrics, such as the DBQ, relies on driver integrity and real-time machine learning application should rather be pursued for reactive instead of remedial results. A thorough review of the current and state of the art driver behaviour applications was performed and it is evident that the areas of driver assistance, intelligent vehicle systems, insurance and theft or hijack detection problems can be addressed and improved in future research. The algorithms applied in the examined studies are categorized and their applications listed, which enables for the identification of probable implementations to replicate and build on in future. Algorithms of specific interest for future investigation are Fuzzy Logic inference systems, HMM and SVM implementations. Difficulties introduced by the large scope of driver behaviour research was overcome by grouping relevant applications together and identifying similar algorithms and techniques used in these applications. Ample opportunity exist to develop unique driver identification systems but complications such as access to necessary sensors and model complexity reduction will need to be addressed in future.
